# Morphometric Investigation of a Species Complex in *Mimosa* Section *Batocaulon* Series *Cordistipulae* (Leguminosae, Caesalpinioideae)

**DOI:** 10.3390/plants14020194

**Published:** 2025-01-12

**Authors:** Janaína G. A. Nascimento, Luciano P. Queiroz, Marlon C. Machado, Cássio van den Berg

**Affiliations:** Departamento de Ciências Biológicas, Universidade Estadual de Feira de Santana, Av. Transnordestina s.n., Feira de Santana 44036-900, Bahia, Brazil; janaina.bot@hotmail.com (J.G.A.N.); lpqueiroz@uefs.br (L.P.Q.);

**Keywords:** canonical variate analysis, cluster analysis, multivariate analysis, species delimitation

## Abstract

*Mimosa* series *Cordistipulae* was created by Barneby in 1991, embracing species diagnosed by their small subshrubby habit and the presence of gland-tipped setae and trimerous flowers. Most species are endemic to Northeastern Brazil, and some possess characters deemed diagnostic which nonetheless overlap, making species identification difficult. Our study aimed to test species circumscriptions and sets of characters that could be applied to unequivocally distinguish the species. Twelve populations (225 individuals) were collected at nine localities, encompassing the Brazilian vegetation types *Caatinga*, *Campos Rupestres* and *Restinga*. Linear measurements of 38 floral and vegetative characters were measured and analyzed using Canonical Variate Analysis and cluster analysis. The first two canonical axes explained 41.4% and 18.9% of the variation and separated two populations of the group recently described as a new species. Vegetative characters are more informative for species delimitation than flower characters, and most groups are distinguished primarily by the number of pinnae pairs, number of leaflets per pinna and length of the leaf rachis. The species displaying the highest morphological similarity are *M. misera*, *M. leptantha* and *M. minarum*. The traditional morphometric approach was capable of objectively dealing with a type of variation that would be difficult to interpret by purely examining herbarium specimens.

## 1. Introduction

*Mimosa* L. with 530 species is one of the largest genera of Leguminosae [1], occurring in Central Mexico, the United States, Central America, South America, Southeast Africa and India [2]. In the 1990s, five sections and 78 series of *Mimosa* were established [3]. In this monograph, *Mimosa* section *Batocaulon* DC. series *Cordistipulae* Barneby includes 13 species: *Mimosa guaranitica* Chodat & Hassl., *M. misera* Benth., *M. morroënsis* Barneby, *M. setuligera* Harms, *M. ulbrichiana* Harms, *M. hirsuticaulis* Harms, *M. borboremae* Harms, *M. minarum* Barneby, *M. brevipinna* Benth., *M. leptantha* Benth., *M. cordistipula* Benth., *M. blanchetii* Benth. and *M. xiquexiquensis* Barneby [3]. More recently, five new species, *M. bahiana* J. Gelma, L.P. Queiroz & Van den Berg, *M. confusa* J. Gelma, L.P. Queiroz & Van den Berg, *Mimosa crassifolia* J. Gelma, L.P. Queiroz & Van den Berg, *Mimosa melosa* J. Gelma, L.P. Queiroz & Van den Berg and *M. rubra* V.F.Dutra & F.C.P. Garcia, were described [4,5].

Species of series *Cordistipulae* are often functionally herbaceous subshrubs. They are seldom more than 1 m high and their branches are covered with a viscid indumentum composed of gland-tipped setae. Prickles are generally absent, stipules are lanceolate, spicules and paraphyllidia are absent and flowers are trimerous. Most species occur in the Brazilian state of Bahia and in other states of Northeastern Brazil such as Pernambuco, Ceará and Piauí, growing in open areas of *Caatinga* vegetation or in sandy soils in *Campo Rupestre* vegetation [3,6]. A comprehensive phylogeny of the genus was presented by [1] based on the *trnD-T* plastid spacer. In their study, 259 species of *Mimosa* were sampled, of which 10 belong to ser. *Cordistipulae*. The series was recovered as monophyletic (corresponding to their “Clade I”) and trimerous flowers indicated a putative synapomorphy for the group.

Taxonomy of series *Cordistipulae* has been mostly based on quantitative characters, including the number of pinnae per leaf, number of leaflets per pinna and measurements of leaf and flower structures. Several of these characters overlap in closely related species, rendering identification a difficult task in some groups of species. This is especially the case of the species related to *M. misera* (hereinafter called the *misera* complex). The species *M. misera* had already been regarded as an “imperfect species” [7] without giving justification for this definition. *M. misera* was also pointed out as an “amorphous” and “undetermined” species [3], and the same author listed *M. cordistipula*, *M. guaranitica*, *M. leptantha*, *M. minarum* and *M. setuligera* as closely related to it. Besides these species, during taxonomic work in herbarium specimens we found an overlap in pinnae length between *M. brevipinna* and *M.setuligera*.

Multivariate analyses have been successfully and widely employed to help untangle species complexes in angiosperms [8,9,10,11,12,13,14,15,16]. In Leguminosae, a search in the literature revealed that many “morphometric” studies are actually numerical taxonomy studies trying to infer species relationships with cluster analyses of qualitative and quantitative characters (e.g., [17,18,19]). However, there are several studies that demonstrated the potential of morphometric data associated with multivariate ordination techniques to help in the delimitation of species complexes in genera taxonomically scattered within the family, such as *Acacia* [20], *Apuleia* [21], *Astragalus* [22], *Bauhinia* [23], *Chamaecrista* [24], *Daniellia* [25], *Daviesia* [26], *Lens* [27], *Lupinus* [28] and *Ononis* [29], among others. Some of these studies included also genetic data, but many obtained good results only with morphological quantitative evidence. Despite this, the number of studies using morphometrics for delimiting species complexes in legumes can be considered small in relation to the size of the family and the abundance of species complexes lacking studies.

In this work we carry out morphometric analyses of putative taxa within ser. *Cordistipulae*, aiming to assess if quantitative linear measurements associated with multivariate analyses can clarify the interspecific boundaries within the *misera* complex to answer the following questions: Which suites of morphological characters are useful for recognizing species within the group? How can morphological variation be evaluated and the species be identified? What are the relationships between morphological variation, species niches and phylogeny of the species? How effective are morphometric methods for dealing with species complexes difficult to resolve with traditional herbarium taxonomy?

## 2. Results

**Figure 1:**
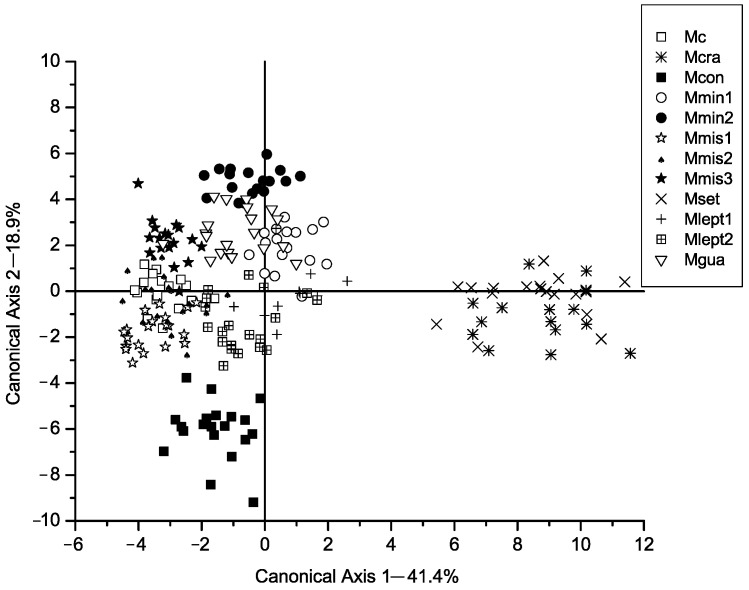
Canonical axis 1 and 2 of a Canonical Variate Analysis of twelve populations in *Mimosa* series *Cordistipulae* based on 38 morphological characters. Axis 1 corresponds to 41.40% of the total variance and axis 2 corresponds to 18.9%. For legend codes, please see Table 3.

**Figure 2:**
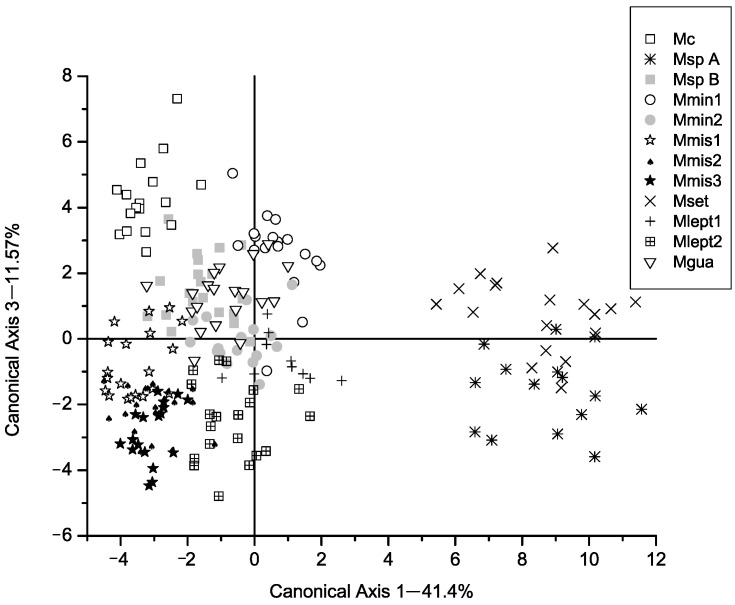
Canonical axis 1 and 3 of a Canonical Variate Analysis of twelve populations in *Mimosa* series *Cordistipulae* based on 38 morphological characters. Axis 1 corresponds to 11.57% of the total variance. Groups separated in previous axes have been grayed out.

**Figure 3:**
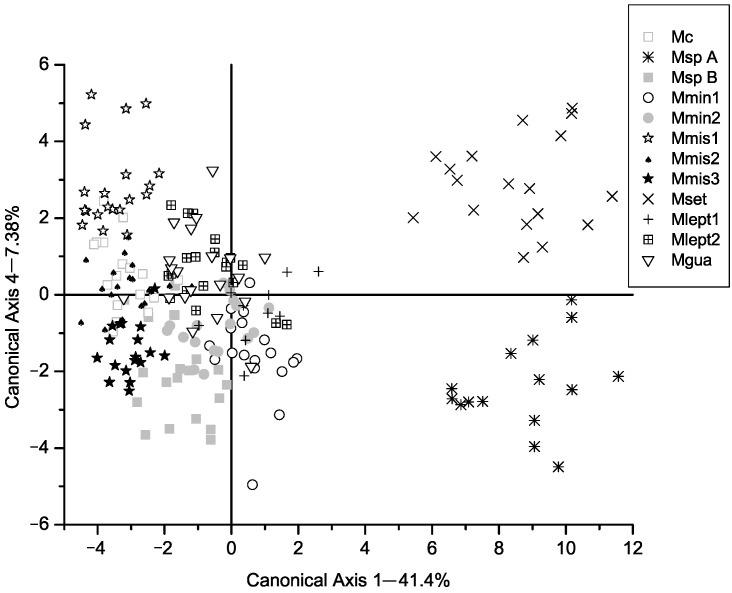
Canonical axis 1 and 4 of a Canonical Variate Analysis of twelve populations in *Mimosa* series *Cordistipulae* based on 38 morphological characters. Axis 1 corresponds to 7.38% of the total variance. Groups separated in previous axes have been grayed out.

All the chi-square values associated with the eleven axes of the CVA were significant (Table 1). In the classification matrix, no individual was classified outside the group to which it was originally assigned. Well-defined groups were formed, as can be observed in Figure 1, Figure 2 and Figure 3, Figures S1 and S2. These groups mostly correspond to species described by Barneby [3,6], except for two groups.

The vegetative characters most important for the differentiation of the species were the number of pinnae per leaf, number of leaflet per pinna, distance between leaflets along the pinna and leaflet width. Among the reproductive characters the most important were stamen length and bracteole dimensions.

The first axis in the scatterplot of Figure 1 explains 41.40% of the total variation. Two groups can be distinguished along the first axis: one includes the populations of *M. setuligera* (Mset) and the recently described *Mimosa crassifolia* (Mcra) and denominated G1, and the second group comprises the remaining populations. The most important characters for the differentiation of these groups were vegetative characters (# V.6, V.11, V.12, V.13, V.21; Table 2). The differentiation of *M. setuligera* and *M*. *crassifolia* from the remaining taxa in G2 is due to them having ca. twice the number of pinnae per leaf and leaflets twice as long as the taxa in G2.

The second axis explains 18.9% of the total variation (Figure 1) and distinguishes the recently described *M. confusa* (Mcon) from other species in group G2. The most important characters for the differentiation of this species are V.14, V.18, V.27 and V.28. In this case *M. confusa* differs from other species in group G2 by having a larger number of leaflets in the terminal pinnae (e.g., double the number of *M. cordistipula*), oblong basal leaflets (linear in the remaining species of G2) and many more flowers (often double) per glomerule (more than 80 flowers per inflorescence).

The third canonical axis (Figure 2) distinguishes *M. cordistipula* (Mc) from the remaining populations, in particular due to the characters V.1, V.6, V.23, V.25 and V.31 (Table 2). These characters indicate a much larger stipule length and petiole length, and a much larger width of the last leaflet pairs for this species. The stipules are much larger in *M. cordistipula*, e.g., in comparison to *M. misera*. In the former, the stipules are persistent, even after dropping leaflets, and measure 4–6 mm. They are deeply lanceolate and have a spinescent apex. In *M. misera*, the stipules are much smaller, caducous with the leaflets, measure 2–4 mm and are never spinescent. In addition to these measures, the terminal leaflets in the first pair of pinnae are half the size in relation to the remaining species in the group.

**Table 1:** Chi-square tests for the successive removal of canonical axes, associated with a Canonical Variate Analysis of 12 populations of various taxa in *Mimosa* series *Cordistipulae* based on 35 linear measurements and meristic variables.

Axis Removed	Eigenvalue	Canonical R	Wilks’ Lambda	*X* ^2^	d.f. *	*p* **
0	16.49	0.971	0.000001	2671.032	418	0.000000
1	8.67	0.947	0.000010	2138.772	370	0.000000
2	6.74	0.933	0.000098	1716.765	324	0.000000
3	4.59	0.906	0.000760	1336.009	280	0.000000
4	3.00	0.866	0.004248	1015.796	238	0.000000
5	2.14	0.825	0.016978	758.105	198	0.000000
6	1.60	0.785	0.053239	545.530	160	0.000000
7	1.21	0.741	0.138549	367.634	124	0.000000
8	0.84	0.677	0.306866	219.730	90	0.000000
9	0.36	0.516	0.566029	105.855	58	0.000127
10	0.30	0.478	0.771238	48.315	28	0.009909

* Degrees of freedom associated with the chi-square value, ** *p*-value relative to the chi-square table at the required d.f.

In the fourth canonical axis (Figure 3) the two species within group G1, *M. setuligera* (Mset) and the recently described *M. crassifolia* (Mcra), are separated, with the most important characters being V.9, V.11, V.24, V.30 and V.38 (Table 2). These variables reflect the well-defined leaf patterns that distinguish these two species. *Mimosa setuligera* presents a larger number of pinnae along the leaf rachis in relation to *M. crassifolia*. Moreover, these pinnae are relatively shorter than *M. setuligera*. Also, the leaflets of *M. setuligera* are spathulate whereas in *M. crassifolia* they are oblong. In this case, floral characters also are important because the stamens of *M. setuligera* are longer, being 7–9 mm, whereas in *M. crassifolia* they are 4–5 mm long.

**Table 2:** Linear measurements and meristic variables for a multivariate morphometrics study of *Mimosa* series *Cordistipulae*.

Variable	Character Description
1	Stipule length (mm)
2	Stipule width at ¾ of the length from the base (mm)
3	Stipule width at ½ of the length from the base (mm)
4	Stipule width at ¼ of the length from the base (mm)
5	Rachis length (mm)
6	Petiole length (including pulvinus) (mm)
7	First interpinal segment length (mm)
8	Last interpinal segment length (mm)
9	Length of the rachis in the pinna of the first pair (mm)
10	Length of the rachis in the pinna of the last pair (mm)
11	Minimum number of pinnae pairs
12	Maximum number of pinnae pairs
13	Number of leaflet pairs in the first pinna
14	Number of leaflet pairs in the last pinna
15	First leaflet pair length (mm)
16	First leaflet pair width in the first pinna at ¾ of the length from the base (mm)
17	First leaflet pair width in the first pinna at ½ of the length from the base (mm)
18	First leaflet pair width in the first pinna at ½ of the length from the base (mm)
19	Length of the third pair of leaflets (mm)
20	Third leaflet pair width in the first pinna at ¾ of the length from the base (mm)
21	Third leaflet pair width in the first pinna at ½ of the length from the base (mm)
22	Third leaflet pair width in the first pinna at ¼ of the length from the base (mm)
23	Length of the last pair of leaflets (mm)
24	Last leaflet pair width in the first pinna at ¾ of the length from the base (mm)
25	Last leaflet pair width in the first pinna at ½ of the length from the base (mm)
26	Last leaflet pair width in the first pinna at ¾ of the length from the base (mm)
27	Peduncle length (mm)
28	Number of flowers per head
29	Bracteole length (mm)
30	Bracteole width at ¾ of the length from the base (mm)
31	Bracteole width at ½ of the length from the base (mm)
32	Bracteole width at ¼ of the length from the base (mm)
33	Calyx length (mm)
34	Corolla tube length (mm)
35	Corolla lobe length (mm)
36	Corolla lobe width at the base (mm)
37	Gynoecium length (mm)
38	Androecium length (mm)

The fifth axis distinguishes *M. guaranitica* (Mgua) from other species, especially *M. leptantha*, and one of the populations of *M. minarum* (Mmin1, Supplementary Figure S1), with the most important characters being V.5, V.7, V.20 and V.31. *Mimosa guaranitica* can therefore be distinguished by its rachis dimensions of the first interpinnae segment. The rachis of *M. guaranitica* is relatively shorter but the interpinnae segment is longer when compared to *M. minarum* and *M. leptantha*. Therefore, pinnae of *M. guaranitica* are more sparsely distributed in relation to the other two species. Additionally, *M. guaranitica* has narrower bracteoles in relation to the other two.

**Table 3:** Sampling locations and vouchers for the populations included in a morphometric study of *Mimosa* series *Cordistipulae.* All locations are in Brazil, and vouchers are deposited at the Herbarium of the Universidade Estadual de Feira de Santana (HUEFS).

Taxon	Voucher/Sample Size	Population Code	Locality Data (State, Municipality, Site)	Geographical Coordinates
*M. confusa*	*Queiroz* et al. *7733* (20)	Mcon	Bahia, Morro do Chapéu, Lajes in the road to Irecê	11°36′58″ S, 41°00′18″ W
*M. cordistipula*	*Nascimento 149* (19)	Mc	Bahia, Morro do Chapéu	11°36′4.7″ S, 41°9′46.6″ W
*M. crassifolia*	*Santos* et al. *355* (15)	Mcra	Bahia, Morro do Chapéu, Tabuleiro dos Tigres	11°36′4.7″ S, 41°9′46.6″ W
*M. guaranitica*	*Santos 807* (20)	Mgua	Bahia, Rio de Contas, Estrada para o Pico das Almas	13°28′12″ S, 41°50′25″ W
*M. leptantha*	*Nascimento 453* (19)	Mlept1	Ceará, Aracati, Cumbi	4°29′34″ S, 37′45″33.2 W
*M. leptantha*	*Nascimento 473* (19)	Mlept2	Rio Grande do Norte, Caraúbas, road to Governador Rosado	5°44′46.9″ S, 37°33′38.7″ W
*M. minarum*	*Nascimento 495* (18)	Mmin1	Minas Gerais, Joaquim Felício, Serra do Cabral	17°41′34″ S, 44°11′56″ W
*M. minarum*	*Nascimento 520* (17)	Mmin2	Minas Gerais, Grão Mogol, 3 km ao N de Grão-Mogol	16°36′47.3″ S, 42°56′S 27.6″ W
*M misera*	*Nascimento 359* (20)	Mmis1	Bahia, Remanso, road to São Raimundo Nonato	9°45′ S, 42°17′ W
*M. misera*	*Lima 177* (20)	Mmis2	Bahia, Canudos, Estação Biológica de Canudos, Base II	10°1′ S, 39°9′ W
*M. misera*	*Nascimento 381* (19)	Mmis3	Piauí, Oeiras, road to Gaturiana	6°58′18.4″ S, 42°1′37″ W
*M. setuligera*	*Nascimento 338* (19)	Mset	Bahia, Remanso, road to São Raimundo Nonato	9°45′ S, 42°18′ W

The sixth axis distinguishes the populations of *M. leptantha* (Mlept1 and Mlept2) from the populations of *M. misera* (Mmis1, Mmis2 and Mmis3), with slight overlaps (Supplementary Figure S2). These are similar species from different habitats (*Restinga* and *Caatinga*). *M. leptantha* is distinguished from *M. misera* by its shorter first interpinnae segments and larger number of pinnae pairs and leaflet pairs per pinna (V.7, V.11, V.14). In this way, the pinnae of *M. leptantha* are more clustered along the rachis, even though the length of the rachis between the two species is similar.

**Figure 4:**
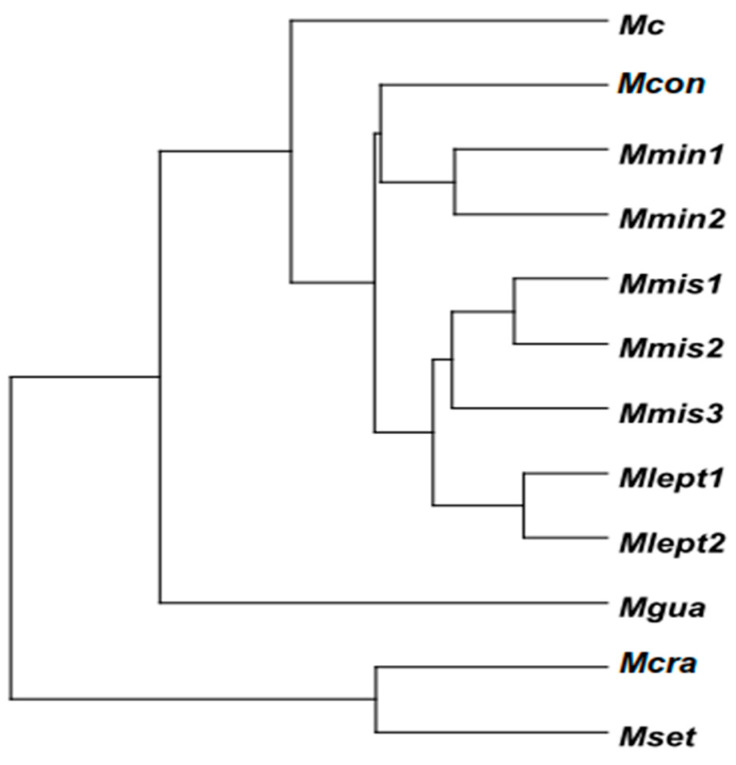
Phenetic relationships among 12 populations of *Mimosa* series *Cordistipulae* based on the Mahalanobis Distance and the Unweighted Pair Group Method with Arithmetic Averages (UPGMA) clustering algorithm on 38 morphological characters. For population codes see Table 3.

Two major groups can be observed in the dendrogram of Figure 4, which correspond to the same groups, G1 and G2, seen in the CDA (Figure 1). The populations assumed to be conspecific are grouped together. The smallest distance is observed among the populations of *M. leptantha*, followed by the populations of *M. misera*, where the most different population is Mmis3 (Oeiras). *Mimosa guaranitica* was clustered near the populations of *M. minarum*. The populations Mcon and Mc are the most distinct within group G2.

## 3. Discussion

### 3.1. Comparison Between Morphometric Patterns and Traditional Taxonomy of the M. misera Species Complex

From the many species concepts used in the literature, population morphometric data rely on the species concept of Templeton [through intrinsic resources of cohesion [30]], in which “a species is represented by the largest group of individuals that have competence of phenotypic cohesion”. This means that we are using the cohesion between individuals within a population to try to evaluate the separation of species; for more discussion on species concepts in morphometrics see [31]. One of the main aims of the present study was to assess the difficult circumscription of *M. misera*, as indicated in the literature. As circumscribed by morphometric data, *M. misera* (occurring in sandy areas of the inland semi-arid *Caatingas*) appeared to be more similar to *M. leptantha* than to *M. guaranitica*, in opposition to the ideas presented in the earlier taxonomic monograph of the group [3]. *M. leptantha* occurs in sand dunes along the coast, in *Restinga* vegetation, whereas *M. guaranitica* occurs in highland *Campo Rupestre* vegetation (also in sandy soils) and disjunctively in savanna vegetation of northeastern Paraguay and Argentina. It also occurs in northeastern Mexico but the latter occurrence has been assumed as a recent invasion [3]: “an antropochorous newcomer to Mexico”. This suggests that probably *M. misera* and *M. leptantha* can be a pair of related species that share similar sandy habitats in lowlands, but originated by allopatric speciation, whereas *M. guaranitica* occurs in much higher habitats. In the UPGMA cluster analysis the populations of *Mimosa misera* group together. This provides evidence that there are characters that unite the populations of this species, at least at the quantitative morphometric level. However, no single diagnostic characters could be identified, and the aggregation of the *M. misera* populations is due to a combination of several vegetative characters, including measurements of the petiole, stipules, rachis, pinnae and leaflets. Beyond this polythetic nature, which in fact extends to many species of the whole group studied, *M. misera* seems to bear the set of characters with the most intermediacy among all others in the group (for this reason it was clearly separated only in axis 6 of the CVA, indicating that there were many other stronger patterns of separation in the group before this one could be observed), which explains why this taxon has been historically regarded as problematic: [6] considered *M. misera* an “imperfect species”, and [3] considered it as “amorphous” and “undetermined”. On the other hand, the results indicate the power of morphometric data together with CDA to recover patterns of quantitative variation that cannot be studied in herbarium materials by pure observation without statistical techniques.

The position of *M. minarum* also deserves attention. Previously, there have been suggested morphological affinities among *M. minarum*, *M. guaranitica* and *M. misera* [3], with the main difference between *M. minarum* and *M. guaranitica* being the petiole length. Our results point again to a combination of characters, such as stipule length, number of pinnae pairs and leaflet measurements. In the phylogeny of *Mimosa* [1], *M. minarum* was not even in the same clade as *M. guaranitica* and *M. misera*. It was sister to a different clade, with species that presented no problems for delimitation. *Mimosa rubra* is morphologically quite similar and occurs in sympatry with *M. minarum*, differing mostly by the reddish branches and foliage and sparse indumentum [4], and is probably phylogenetically related to *M. minarum*. The morphometric and phylogenetic differences have additional support from its allopatric distribution. *Mimosa minarum* occurs in Minas Gerais state, at least 600 km away from populations of *M. guaranitica* and *M. misera*.

### 3.2. Overall Comparison Between Morphometric Clusters and Phylogenetic Relationships

A phylogenetic study of *Mimosa* [1] sampled 10 out of the 18 species ascribed to ser. *Cordistipulae*. The series was strongly supported as monophyletic with two well-supported subclades. Species of the *misera* complex appeared in the two subclades: (A) *M. minarum* and *M. setuligera* grouped with *M. blanchetii* and *M. xiquexiquensis* and (B) *M. cordistipula*, *M. guaranitica*, *M. leptantha* and *M. misera* clustered with *M. morroënsis* and *M. ulbrichiana*. The fact that species of the *misera* complex appeared dispersed in two well-supported monophyletic groups is a clear indicator that the similar morphology found in the *misera* complex arose by convergence rather than as a reflection of common ancestry. The subclade A brings together rupicolous species that grow in high mountain areas above 900 m, except for the lowland sand dune *M. xiquexiquensis*, while subclade B holds lowland species that grow until 600 m high. This finding is in agreement with the general phylogenetic pattern found in *Mimosa* [1], in which the deeper nodes in *Mimosa* phylogeny agree better with geographical areas than morphology. At lower levels, ecology seems to better explain relationships than morphology within ser. *Cordistipulae*.

### 3.3. Usefulness and Perspectives for the Use of Multivariate Morphometrics in Leguminosae

When trying to compare the performance of our study with other morphometric studies in Leguminosae, we verified the extreme heterogeneity of approaches and purposes. As an example, in the present study vegetative characters performed clearly better at differentiating species than floral characters. This seems to be a common pattern in legumes, at least at the morphometric level. Often, species taxonomy in Leguminosae relies heavily on leaf characters, and there is extensive variation in leaflet size among individuals within and between populations. This size variation as well as allometric effects in shape could explain the difficulties of previous *Mimosa* taxonomists in interpreting the species patterns that seem quite evident in the results of our analyses. For this reason, we should stress here the usefulness of multivariate morphometrics to deal with species complexes where the standard herbarium approach does not achieve immediate success, and species seem to overlap. Another morphometric study trying to delimit species of *Chamaecrista* [24] also pointed out the use of leaf characteristics, when the number of leaflet pairs, the length of the second pair of leaflets and petiole length provided well-delimited taxa. Ours and the latter study had a very similar experimental design, with multiple population samples taken within species and multiple samples per population. Despite the fact that multivariate morphometrics is composed of old and straightforward techniques, very few studies follow the same experimental design as that just mentioned. After an extensive review, we found that early studies tended to try to infer the “relationships” of a group of species based on a few samples of each (e.g., [17,31]) in the old numerical taxonomy context, which is considered today a bad use for morphometric data. Other studies were mostly interested in the separation of a single pair of species [32,33,34,35,36], with reasonable success. Some other studies used a mixture of genetic and morphometric data, generally sampling a single population or even pooling scattered individuals of each species and trying to detect differences [20,21,37,38]. This type of approach should rely on analyses that do not imply previous group assumptions (e.g., Principal Components of Principal Coordinates). Because of the statistical assumptions, the use of CVA (as in our study) should be carried out with caution. To use this technique, the experimental design must sample multiple individuals in real populations in the field and try whenever possible to have multiple populations of the assumed taxa (though for some endemic taxa this will not be possible, as in the case of some species in the current study). The algebra involved in CVA is based on removing the variation in the within-population variance followed by maximization of the group differences [37,38,39]. If CVA is applied to an experimental design with only a single population of each species, or, worse, a pooled collection of herbarium specimens from different locations representing each species, the results could be philosophically flawed [40]. In this case, the variation within each species will be shrunk by the statistical technique, and differences and discontinuities might be simply caused by the groups assigned prior to the analysis, rather than any real biological differences. In this type of experimental design, researchers should prefer to base their results only on Principal Component Analysis (PCA) instead of CVA. The PCA approach should be used in this experimental design to preserve the original distances between samples, and if distinct groups are recovered in the analysis they will correspond to real discontinuities that might be interpreted as putative species. Based on the results of the current study and the optimal experimental design for CVA, we suggest that future studies aiming at species delimitation are performed always based on an experimental design previously intended for multivariate morphometric analysis of population data, with individual sampling of the populations in each location, multiple locations per species whenever possible and the use of CVA. Besides the current study, there was only one other study with field sampling designed specifically for CVA that we found and cited in the current paper [24]. When based only on pooled herbarium samples for each species, the studies should avoid CVA and prefer PCA, PCO and NMDS despite their lower discriminatory power (good examples of this design in legumes are [22,41,42]).

## 4. Materials and Methods

### 4.1. Data Collection

**Figure 5:**
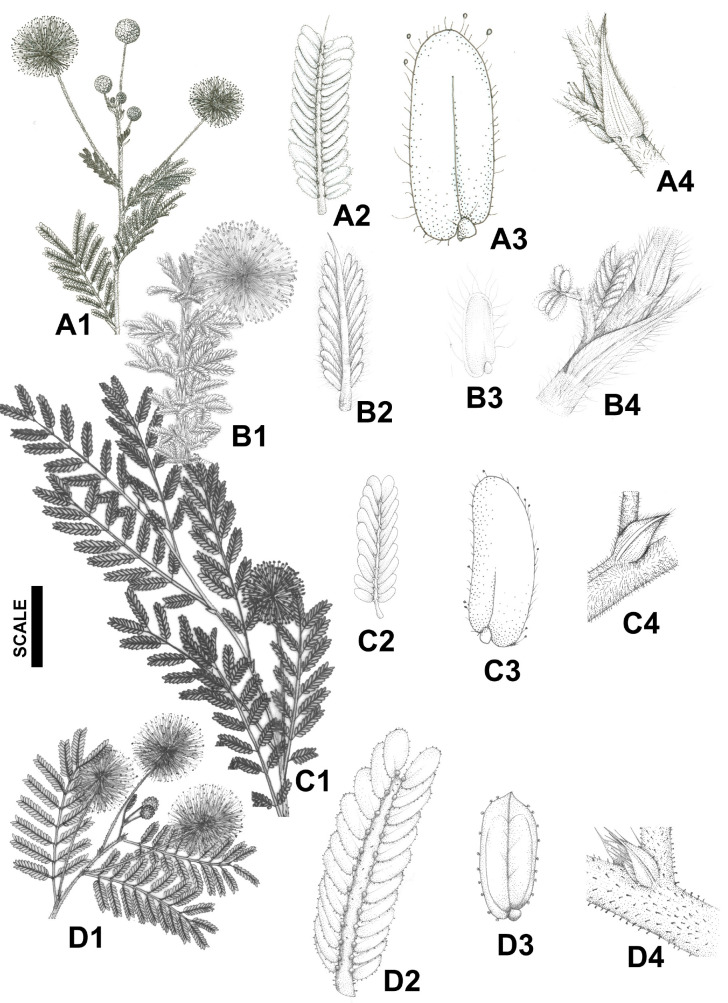
Morphology of species of *Mimosa* ser. *Cordistipulae*. The same structures are represented for all species. *Mimosa confusa*: flowering branch (**A1**), one pinna (**A2**), leaflet (**A3**), stipule (**A4**); *Mimosa cordistipula*: flowering branch (**B1**), one pinna (**B2**), leaflet (**B3**), stipule (**B4**); *Mimosa guaranitica*: flowering branch (**C1**), one pinna (**C2**), leaflet (**C3**), stipule (**C4**); and *Mimosa crassifolia*: flowering branch (**D1**), one pinna (**D2**), leaflet (**D3**), stipule (**D4**). All structures are drawn to the same scale: flowering branches, scale = 20 mm (**A1**,**B1**,**C1**,**D1**); pinnae, scale = 8.5 mm (**A2**,**B2**,**C2**,**D2**); leaflets, scale = 2.1 mm (**A3**,**B3**,**C3**,**D3**); and stipules, scale = 3.3 mm (**A4**,**B4**,**C4**,**D4**). Drawings are by Carla de Lima.

**Figure 6:**
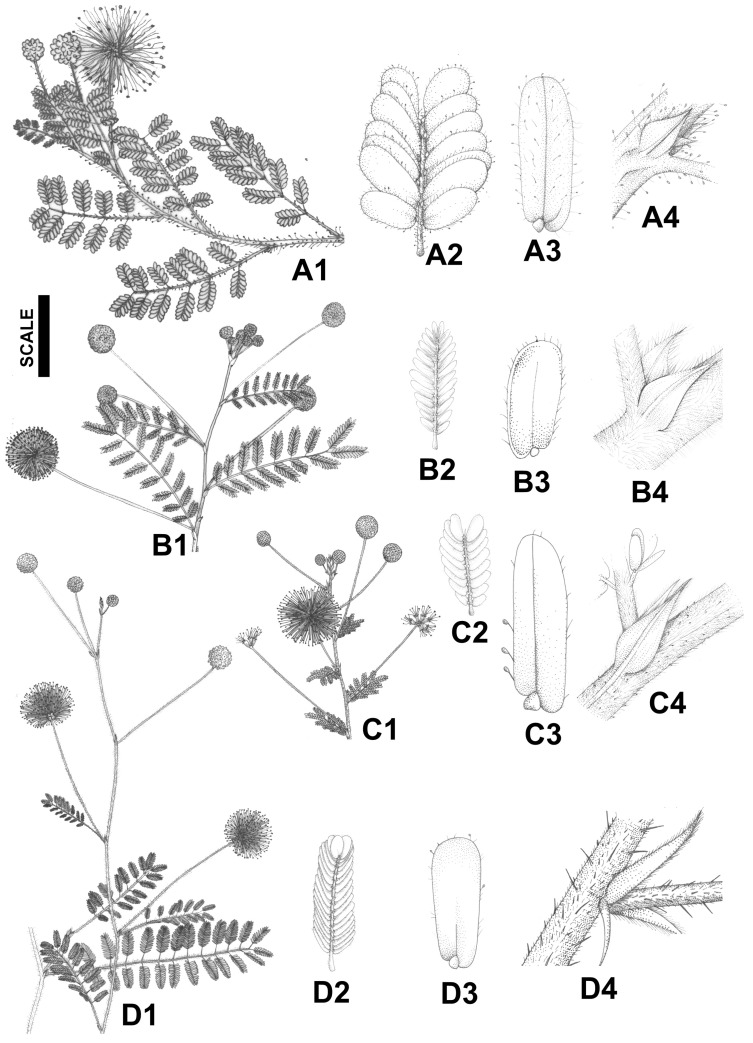
Morphology of species of *Mimosa* ser. *Cordistipulae*. The same structures are represented for all species. *Mimosa leptantha*: flowering branch (**A1**), one pinna (**A2**), leaflet (**A3**), stipule (**A4**); *Mimosa minarum*: flowering branch (**B1**), one pinna (**B2**), leaflet (**B3**), stipule (**B4**); *Mimosa misera*: flowering branch (**C1**), one pinna (**C2**), leaflet (**C3**), stipule (**C4**); and *Mimosa setuligera*: flowering branch (**D1**), one pinna (**D2**), leaflet (**D3**), stipule (**D4**). All structures are drawn to the same scale: flowering branches, scale = 20 mm (**A1**,**B1**,**C1**,**D1**); pinnae, scale = 8.5 mm (**A2**,**B2**,**C2**,**D2**); leaflets, scale = 2.1 mm (**A3**,**B3**,**C3**,**D3**); and stipules, scale = 3.3 mm (**A4**,**B4**,**C4**,**D4**). Drawings are by Carla de Lima.

**Figure 7:**
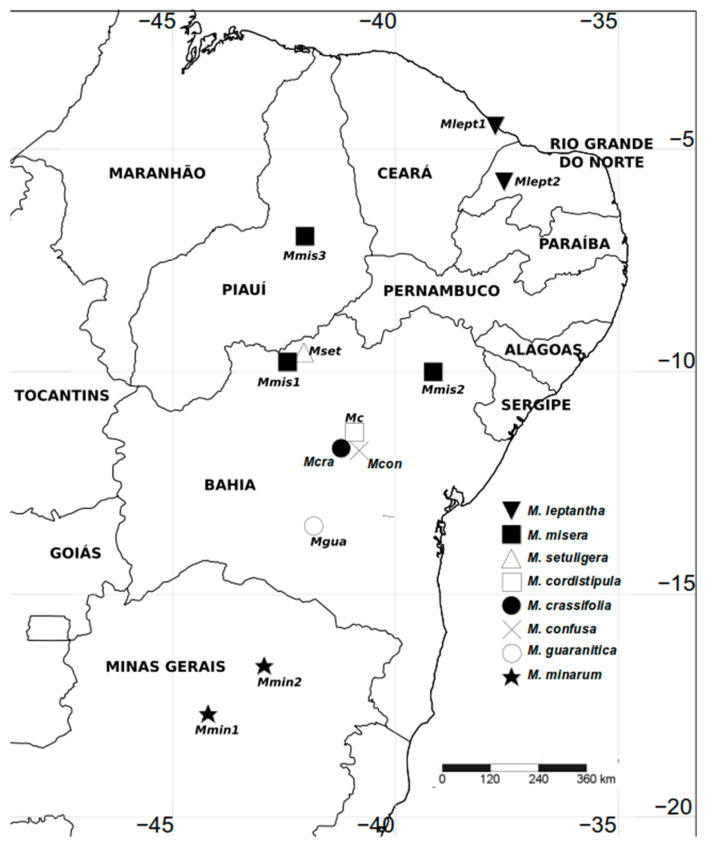
Sampling locations for the populations included in a morphometric study of *Mimosa* series *Cordistipulae*. Population codes are given in Table 3.

We sampled a total of 225 individuals from eight species (Figure 5 and Figure 6) in 12 natural populations (Table 3; Figure 7), including all taxa of the *misera* complex, encompassing the entire range of the species, including type localities, except in the case of *M. guaranitica*, for which it was not possible to sample extra-Brazilian populations, and the rare and newly described *M. rubra*, which is known by only by two collections [4] and was described after we finished our experiment. Also, the species *Mimosa bahiana* and *M. melosa*, recently described in [5] from herbarium material, were not known when we collected the data used in the current study, and the available herbarium material was unsuitable for the statistical designed that was used, which follows. Fifteen-to-twenty individuals were sampled at each population, and the individuals collected were at least one meter apart from each other, to avoid clonality. The definition of the sampling area was based on the geographical distribution of series *Cordistipulae*, which is highly concentrated in Eastern Brazil, in the northeastern coast, from Ceará to Rio Grande do Norte states (3°58′ S) to northern Minas Gerais state (17°45′ S). Populations were assigned to species recognized by Barneby [3] using diagnostic characters proposed by this author. As we aimed to test species circumscription, the ascription of a population to a species represented only an initial taxonomic hypothesis, but was not used as a categorial variable for the analyses. We collected one population of *M. cordistipula* (Mc), *M. guaranitica* (Mgua) and *M. setuligera* (Mset), two populations of *M. lepthanta* (Mlept1, Mlept2) and *M. minarum* (Mmin1, Mmin2) and three populations of *M. misera* (Mmis1, Mmis2, Mmis3). Two populations included in this study could not be assigned to any known species at the time of data collection but were later described with the names *Mimosa crassifolia* (Mcra) and *Mimosa confusa* (Mcon) [40], based on the data that are presented in the current paper. *M. brevipinna* (known only from the type of material deposited at K) was not found in the field, despite efforts made in several field trips, and therefore could not be included in this study. Vouchers are housed at the herbarium of Feira de Santana State University (HUEFS; Table 3). A total of 38 linear measurements were taken, comprising 12 floral characters and 26 vegetative characters (Table 2). Measures were taken from the third leaf from the apex and from the apical flowers of the apical glomerule of axillary inflorescences. Due to the small size of the structures, measures of continuous characters were taken from camera lucida outlines of the structures using a Zeiss Stemi SV11 stereomicroscope. The measurements of the drawings were made with an engineer’s scale and then converted back to the real sizes of the structures. A data matrix with all the measurements was prepared for the statistical analyses.

### 4.2. Data Analysis

We employed Canonical Variate Analysis (CVA), taking only the populations as a categorical variable (the individuals were grouped according to the populations to which they belong) in order to detect the patterns of differentiation of groups and evaluate which characters influence their separation, without pre-defining the species. This could not be avoided in species with a single population; however, in the overall dataset we assessed the clustering and ordination patterns without forcing any of the species clustering by maintaining only the population level as an a priori assumption. The number of axes to be interpreted was chosen based on the chi-square value obtained from successive root removal [43]. The standardized coefficients were used to analyze the contribution of characters to canonical axes. A cluster analysis was performed in order to investigate patterns of hierarchical clustering of the different groups. For this purpose, we used the UPGMA algorithm (Unweighted Pair Group Method with Arithmetic Averages [44]) on the Generalized Mahalanobis Distances between group centroids obtained during the CVA. In the CVA we also examined the classification matrix of individuals in the groups, based on the proximity to the inferred centroids for each group. This was used to assess the level of coherence of each group originally postulated.

## Data Availability

Original data matrices can be requested from the C.v.d.B.

## Supplementary Materials

The following supporting information can be downloaded at https://www.mdpi.com/article/10.3390/plants14020194/s1. Figure S1. Canonical Axis 1 and 5 of a Canonical Variate Analysis of twelve populations in *Mimosa* series *Cordistipulae* based on 38 morphological characters. Axis one corresponds to 5.92% of the total variance. Groups separated in previous axes have been grayed out. Figure S2. Canonical Axis 1 and 6 of a Canonical Variate Analysis of twelve populations in *Mimosa* series *Cordistipulae* based on 38 morphological characters. Axis one corresponds to 3.76% of the total variance. Groups separated in previous axes have been grayed out.
