# Supplementary figures and images for: Morphometric Investigation of a Species Complex in Mimosa Section Batocaulon Series Cordistipulae (Leguminosae, Caesalpinioideae)
Source: Plants (Basel). 2025 Jan 12;14(2):194. doi: 10.3390/plants14020194 (PMC11769148; doi:10.3390/plants14020194)

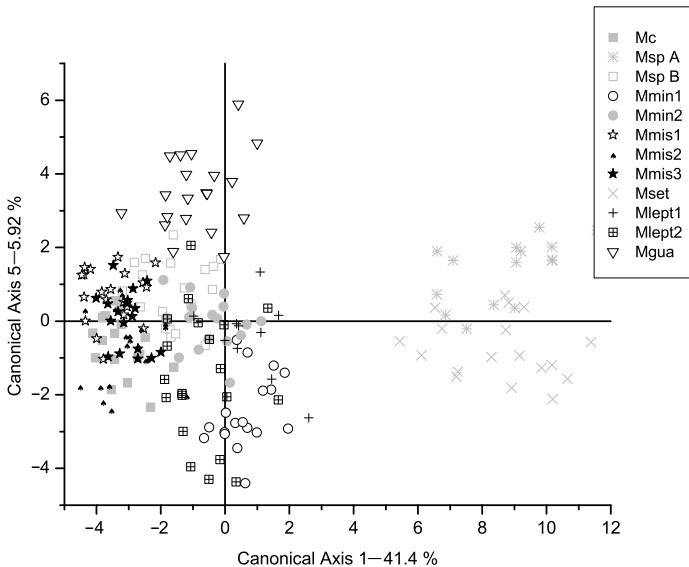

Supplement: Supplementary file 1 [file plants-14-00194-s001.zip › Figure S1.pdf]

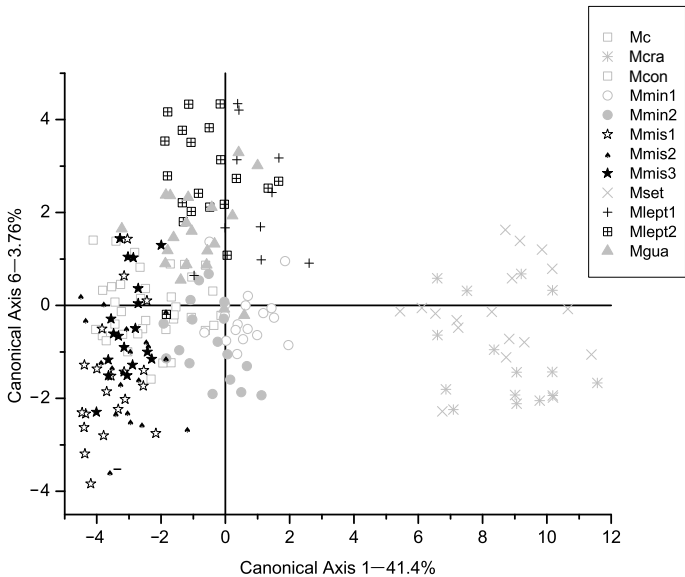

Supplement: Supplementary file 1 [file plants-14-00194-s001.zip › Figure S2.pdf]
